# Complete mitochondrial genome sequence of a xerophilic fungus, *Aspergillus pseudoglaucus*

**DOI:** 10.1080/23802359.2019.1586468

**Published:** 2019-07-12

**Authors:** Jongsun Park, Woochan Kwon, Xiaoxiao Huang, Anbazhagan Mageswari, In-Beom Heo, Kap-Hoon Han, Seung-Beom Hong

**Affiliations:** a InfoBoss Co., Ltd., Seoul, Republic of Korea;; b InfoBoss Research Center, Seoul, Republic of Korea;; c Department of Pharmaceutical Engineering, Woosuk University, Wanju, Republic of Korea;; d Department of Microbiology, D.G. Vaishnav College, Chennai, India;; e Agricultural Microbial Division, National Institute of Agricultural Scinece, Wanjugun, Republic of Korea

**Keywords:** *Aspergillus pseudoglaucus*, mitochondrial genome, a xerophilic fungus, Ascomycota

## Abstract

*Aspergillus pseudoglaucus* is a xerophilic filamentous fungus which can produce various secondary metabolites. Here, we reported the complete mitochondrial genome sequence of *A. pseudoglaucus* isolated from Meju, a soybean brick in Korea. Its mitochondrial genome was successfully assembled from raw reads sequenced using MiSeq by Velvet and SOAPGapCloser. Total length of the mitochondrial genome is 53,882 bp, which is third longest among known *Aspergillus* mitochondrial genomes and encoded 58 genes (30 protein-coding genes including hypothetical ORFs, two rRNAs, and 26 tRNAs). Nucleotide sequence of coding regions takes over 66.6% and overall GC content is 27.8%. Phylogenetic trees present that *A. pseudoglaucus* is located outside of section *Nidulantes*. Additional researches will be required for clarifying phylogenetic position of section *Aspergillus*.

*Aspergillus pseudoglaucus* (Chen et al. 2017), identified in 1929, is a xerophilic filamentous fungus belonging to *Aspergillus* section *Aspergillus*. It has been isolated from seafoods and soil (Smetanina et al. 2007; Séguin et al. 2014). It has also been used as a start culture for Kastuobushi in Japan (Pitt and Hocking 2009) and frequently identified from Meju, a soybean brick used for making soy source and soybean paste in Korea (Hong et al. 2011). It can produce various metabolites such as benzyl derivatives binding to human opioid or cannabinoid receptors (Gao et al. 2011), mycophenolic acid (Mouhamadou et al. 2017), and various antibacterial and antifungal compounds (Gao et al. 2012). To understand phylogenetic relationship of *A. pseudoglaucus*, we presented its complete mitochondrial genome.

DNA of *A. pseudoglaucus* collected from Meju in Korea was extracted using DNeasy Plant Mini Kit (QIAGEN, Hilden, Germany). Raw data generated using MiSeq, and *de novo* assembly was conducted using Velvet 1.2.10 (Zerbino and Birney 2008). Gap filling was carried out using SOAPGapCloser 1.12 (Zhao et al. 2011) after confirming each base using BWA 0.7.17 and SAMtools 1.9 (Li et al. 2009; Li 2013). Geneious R11 11.0.5 (Biomatters Ltd, Auckland, New Zealand) was used to annotate its mitochondrial genome by comparing with those of *Aspergillus luchuensis* (MK061298; Park, Kwon, Zhu, Mageswari, Heo, Han, Hong, under review) and *Aspergillus parasiticus* (MK124769; Park et al. under review). Voucher sample was deposited into Korean Agricultural Culture Collection (KACC; Republic of Korea; http://genebank.rda.go.kr/; KACC-93211).

The length of *A. pseudoglaucus* mitochondrial genome (Genbank accession is MK202802) is 53,882 bp, which is third longest among twelve *Aspergillus* mitochondrial genomes (Futagami et al. 2011; Joardar et al. 2012; Xu et al. 2018; Park, Kwon, Zhu, Mageswari, Heo, Han, Hong, under review; Park et al. under review): The longest is *Aspergillus cristatus* (77,649 bp; Ge et al. 2016) and second longest is *Aspergillus egyptiacus* (66,526 bp; Xu et al. 2018). *Aspergillus pseudoglaucus CO1* gene contains eight introns, which is proportion to the length of whole mitochondrial genomes (Joardar et al. 2012). In addition, other main protein-coding genes except ND5 have at least one intron, indicating a reason of expansion of *A. pseudoglaucus* mitochondrial genome (Joardar et al. 2012). Moreover, 16 of 20 introns contain partial ORFs, mostly endonuclease. Its mitochondrial genome encoded 58 genes consisting of 30 protein-coding genes including hypothetical ORFs, two rRNAs, and 26 tRNAs. Protein-coding regions takes over 66.6% on genome, and overall GC content is 27.8%, which are similar to those of *Aspergillus* genus.

Sequence alignment of nine *Aspergillus* except *A. cristatus, A. egyptiacus*, and *A. flavus* and one *Penicillium* mitochondrial genome as an outgroup was conducted using MAFFT 7.388 (Katoh and Standley 2013). The neighbor joining (10,000 bootstrap repeats) and maximum likelihood (1,000 bootstrap repeats) methods were used for constructing phylogenetic tree using MEGA X (Kumar et al. 2018). Phylogenetic trees showed that *A. pseudoglaucus* is located outside of section *Nidulantes* and does not form any clades with other species; however, bootstrap supports are not enough (Figure 1). Additional researches will be required for clarifying phylogenetic position of section *Aspergillus*.

**Figure 1.**
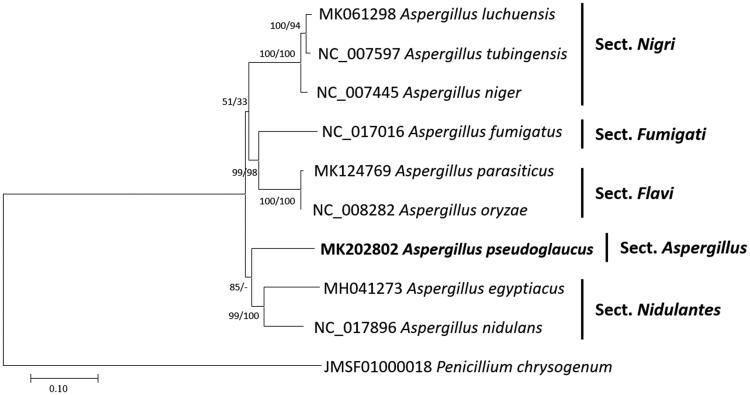
Neighbor joining (bootstrap repeat is 10,000) and maximum likelihood (bootstrap repeat is 1,000) phylogenetic trees of nine *Aspergillus* and one *Penicillium* mitochondrial genome: *Aspergillus pseudoglaucus* (MK202802, this study), *Aspergillus parasiticus* (MK124769), *Aspergillus luchuensis (*MK061298), *Aspergillus egyptiacus* (MH041273), *Aspergillus tubingensis* (NC_007597), *Aspergillus nidulans* (NC_017896), *Aspergillus niger* (NC_007445), *Aspergillus oryzae* (NC_008282), *Aspergillus fumigatus* (NC_017016), and *Penicillium chrysogenum* (JMSF01000018). The numbers above branches indicate bootstrap support values of neighbor joining and maximum likelihood phylogenetic trees, respectively.
